# 10 Hz rhythmic stimulation modulates electrophysiological, but not behavioral markers of suppression

**DOI:** 10.3389/fpsyg.2024.1376664

**Published:** 2024-05-20

**Authors:** Bence Szaszkó, Martin Habeler, Marlene Forstinger, Ulrich Pomper, Manuel Scheftner, Moritz Stolte, Markus Grüner, Ulrich Ansorge

**Affiliations:** ^1^Department of Cognition, Emotion, and Methods in Psychology, University of Vienna, Vienna, Austria; ^2^Vienna Cognitive Science Hub, University of Vienna, Vienna, Austria; ^3^Research Platform Mediatised Lifeworlds, University of Vienna, Vienna, Austria

**Keywords:** alpha oscillations, cueing, entrainment, suppression, visual attention, visual search

## Abstract

We investigated the role of alpha in the suppression of attention capture by salient but to-be-suppressed (negative and nonpredictive) color cues, expecting a potential boosting effect of alpha-rhythmic entrainment on feature-specific cue suppression. We did so by presenting a rhythmically flickering visual bar of 10 Hz before the cue - either on the cue’s side or opposite the cue -while an arrhythmically flickering visual bar was presented on the respective other side. We hypothesized that rhythmic entrainment at cue location could enhance the suppression of the cue. Testing 27 participants ranging from 18 to 39 years of age, we found both behavioral and electrophysiological evidence of suppression: Search times for a target at a negatively cued location were delayed relative to a target away from the cued location (inverse validity effects). In addition, an event-related potential indicative for suppression (the Distractor Positivity, Pd) was observed following rhythmic but not arrhythmic stimulation, indicating that suppression was boosted by the stimulation. This was also echoed in higher spectral power and intertrial phase coherence of EEG at rhythmically versus arrhythmically stimulated electrode sites, albeit only at the second harmonic (20 Hz), but not at the stimulation frequency. In addition, inverse validity effects were not modulated by rhythmic entrainment congruent with the cue side. Hence, we propose that rhythmic visual stimulation in the alpha range could support suppression, though behavioral evidence remains elusive, in contrast to electrophysiological findings.

## Introduction

Visuospatial attention denotes the ability to select visual information from specific locations while ignoring information elsewhere. It is well established that visuospatial attention co-varies with the power of neural oscillations in the alpha-band (8–12 Hz; Suffczynski et al., 2001; Fries et al., 2008; Klimesch, 2012; Pomper and Chait, 2017). For example, sampling visual information from two alternative locations shows rhythmically fluctuating accuracy in the theta- to alpha range, between 4 and10 Hz (Landau and Fries, 2012; Fiebelkorn et al., 2013). Likewise, measures of brain activity showed that decreases in lateralized alpha activity contralateral to the attended-to location correlated with increased attentional performance (Thut et al., 2006; Bauer et al., 2012; Foster and Awh, 2019). One underlying reason could be the improved signal-to-noise ratio of location-specific activity for a relevant target against a silenced background of suppressed activity from neurons representing surrounding locations and alternative distractor stimuli (see also Pfurtscheller et al., 1996; Klimesch et al., 2007; Jensen and Mazaheri, 2010; Foxe and Snyder, 2011; Klimesch, 2012; Wöstmann et al., 2016). One proposed mechanism is the interaction of alpha oscillations with other frequencies, such as gamma-oscillations, by cross-frequency coupling that leads to enhanced timing of information transfer by facilitating the integration of relevant information while suppressing noise (Klimesch et al., 2007). Furthermore, inhibitory activity, frequently reflected in oscillatory alpha activity, could silence brain areas that otherwise interfere with task-relevant processing. Hence, increased performance is achieved via functional inhibition of task-irrelevant areas and pathways, gating information flow in task-relevant areas (Jensen and Mazaheri, 2010). In line with the assumption of an improved signal-to-noise ratio, researchers observed that increased alpha activity contralateral to an invalidly cued location correlated with lower target detection performance at the unattended target location (Händel et al., 2011).

However, the inhibitory role of alpha in visuospatial tasks is still contested as all evidence can be explained by facilitated selection alone, without recursion to (additional) suppression (Foster and Awh, 2019; Van Moorselaar and Slagter, 2020). For example, one problem with findings such as that of Händel et al. (2011) is that it is notoriously difficult to discriminate between alpha decrements ipsilateral to the attended location and alpha increments contralateral to the target position. Thus, higher alpha activity contralateral to the target under invalidly cued conditions could as well reflect ipsilateral effects of attention to the cued location (e.g., difficulties in suppressing the cued location) rather than facilitated suppression of the target position. Thus, it is not clear if and how such alpha activity influences stimulus suppression. A similar argument holds regarding studies using related protocols, such as retrieval of information from competing opposite sides (see also Waldhauser et al., 2012).

### Current tests of suppression

Contributing to this debate, here we investigated the role of alpha amplitude, as well as alpha-phase coherence, in suppression with the help of salient target-preceding cues that were uninformative of the target location. Above all, we were interested to see how visual alpha-rhythmic entrainment as a way to boost alpha activity might affect performance when using salient but spatially uninformative cues carrying a “negative” color – that is, a color that was to be suppressed as that of a task-relevant distractor during target search. Essentially, per each trial, participants had to search for a non-red target and, thus, had to suppress the negative color red of an otherwise target-similar distractor. Past research has shown that the intention to suppress the negative color red already applies to a target-preceding cue of that color, reflected in delayed search times for a target presented at the negatively cued location (under valid conditions) compared to a target presented away from the negative cue (under invalid conditions) (Forstinger et al., 2022). This contrasts with cues in entirely irrelevant colors (e.g., blue cues) in a control condition: They did not create a cost for targets presented at a valid location (Forstinger et al., 2022). In a pilot study leading up to the present experiment, employing both red color singletons as negative cues as well as gray orientation singletons as irrelevant cues, only the former negative cues led to an inverse cueing effect, with no performance differences between valid and invalid trials for the latter irrelevant cues. These results corroborate the findings of Forstinger et al. (2022), indicating that suppression of a feature below baseline is different from its mere ignorance.

To validate whether suppression took place in the current experiment, we, thus, used a similar visual search task, with an uninformative cue preceding each target display, during which participants had to search for a predefined target. We measured suppression with red negative cues, carrying a feature that was absent in the following targets that were conjunctively defined by the presence of a positive feature at the target and the absence of a negative feature, which was only present at the target-similar distractor. Hence, we expected to find a similar suppressive mechanism at work here, slowing down performance in valid compared to invalid conditions. Additionally, we used electrophysiology to corroborate behavioral evidence of suppression by looking at a commonly used indicator of suppression (Hickey et al., 2009), an event-related potential (ERP) labeled *distractor positivity* (Pd). The Pd is a positive deflection contralateral to the object of suppression and reflects alpha lateralization in occipital cortex, visible 100–400 ms (but in paradigms like ours, mostly 115–225 ms) after stimulus onset (Sawaki et al., 2012). However, if or under what conditions the Pd is the first response elicited by a to-be-suppressed stimulus is the subject of intense debate (Hickey et al., 2009; Burra and Kerzel, 2014; Gaspelin and Luck, 2018a, 2018b; Wang et al., 2019; Forschack et al., 2022). Here, we looked for a cue-elicited Pd as the least contaminated evidence of such suppression (for details, see Methods section).

### Manipulation of alpha

Importantly, we expected that alpha oscillations modulated suppression of the negative cue. To test the impact of alpha on feature-specific suppression (of the negative cue), we used rhythmic visual stimulation that is said to lead to entrainment. Neuronal entrainment denotes the temporal alignment of neuronal oscillatory activity to an external stimulus by altering the timing of neuronal excitability (Lakatos et al., 2019). This process enables neuronal ensembles to serve as functional networks and enhances communication between cortical areas (Fries, 2005; Bastos et al., 2015). Here, the entrainment stimulus consisted of a visual bar rhythmically flickering at a fixed alpha frequency (of 10 Hz) on one side (e.g., on the left) of the display. It was presented prior to the cue and target displays and accompanied by a visual bar flickering non-rhythmically, with more variable period lengths between successive luminance peaks on the opposite side (Spaak et al., 2014). In comparison to the usage of electrophysiological recordings without external visual stimulation (e.g., Busch et al., 2009), the usage of an alpha-entrainment stimulus has the advantage of allowing us to manipulate endogenous alpha that aligns its phase with the external stimulation rather than having to rely on alpha’s naturally occurring fluctuations in brain activity to sort trials post-measurement into alpha-positive (or more alpha activity) and alpha-negative (or less alpha activity) conditions.

If rhythmic visual stimulation entrained participants’ visual cortex more than arrhythmic stimulation, this difference would have been visible during the period of entrainment when looking at participants’ neuronal activity in the time-frequency domain. Additionally, if alpha boosted suppression in a spatially selective way, we expected negatively cued targets presented on the same side as the preceding rhythmically flickering alpha bars to be more suppressed than negatively cued targets presented on the entrainment-incongruent side. On the behavioral level, this would be present as increased inverse cueing effects on the congruent side, while on the electrophysiological level, it could be visible as an increased cue-elicited Pd.

## Method

### Participants

Using G*power (Faul et al., 2009), a required sample size of 24 participants was determined to achieve 80% power at a significance level of 0.05 and an effect size of Cohen’s *d* = 0.6 (for a two-sided one-sample t test; the effect size was adjusted for repeated measures designs). We opted for the one-sample *t* test for two reasons: First, we intended to investigate differences between rhythmic and non-rhythmic visual stimulation (reflected in the interaction between cue side and entrainment side, see below). Second, we aimed to find corroborating evidence obtained in past experiments (Forstinger et al., 2022) for suppression through negative cues, which could be demonstrated through inverse validity effects (better performance in invalid than in valid trials). Inverse validity effects then can be compared between conditions with a simple *t* test for possible differences in suppression (or capture). Although effects in contingent-capture experiments, where one cue carries features matching an attentional control setting (here, the negative cue matching to a setting to suppress the negative distractor color) tend to be large, with effect sizes often exceeding *d* = 1 – in fact, Büsel et al. (2020), in their meta-analysis, found an effect size of *g* = 1.78 –, the common effect size is not equally well established for inverse validity effects created by negative features. Therefore, we used a medium effect size as a basis for our calculations of a necessary sample size.

Twenty-seven healthy, right-handed psychology students (22 female, ranging from 18 to 39 years of age, *M_age_* = 21.6 years, *SD_age_* = 4.2 years) of the University of Vienna participated in the experiment in exchange for partial course credit. We tested for outliers in the mean accuracy rates using a generalized extreme Studentized deviate test sensitive for multiple outliers (we tested for two outliers; Rosner, 1983), but did not exclude any of the participants. Thus, all 27 participants were included in further data analysis. All participants had normal or corrected-to-normal visual acuity and normal color vision, assessed by self-report and Ishihara color plates (Clark, 1924), respectively. Before the experiment, participants gave written informed consent and received a short introduction to the experimental procedure. After the experiment, participants received written and verbal debriefing. We adhered to the Austrian Universities Act, 2002 (UG2002, Article 30 § 1), according to which only medical universities or studies conducting applied medical research must obtain additional approval by an ethics committee. Thus, no additional ethical approval was required for the present study.

### Apparatus

We conducted the experiment in a dimly lit room. Stimuli were presented on a 19” CRT monitor (Sony Multiscan), with an aspect ratio of 4:3, a resolution of 1,920 × 960 pixels, and a refresh rate of 100 Hz. A chin- and forehead rest ensured a constant viewing distance of 57 cm. The experiment was programmed and executed in PsychoPy 2021.2.0 (Peirce et al., 2019).

### Definition of to-be-suppressed features

We defined the color of the negative cue by our visual search task. In target-present trials, we used *T* junctions of two oriented lines as targets. These targets were conjunctively defined by the presence of a positive color (a blue vertical base line) and the absence of a to-be-suppressed or negative color, here, a particular distractor color (a red horizontal line, Forstinger et al., 2022). Critically, participants had to suppress the negative color to find the target, as (1) two blue lines of the target’s orientation were present in two different stimuli per target display – one in the target itself and one in the negative distractor – and (2) there was no second positive feature that could be used consistently to search for the target. The latter was achieved by endowing the second target line with one out of three randomly changing colors (cyan, magenta, or gray). In each target-search display, only one of these colors was used for the target. Critically, the other two potential target colors (e.g., magenta and gray, if the current target’s second line was cyan) were used for the coloration of two further *T* junction distractors in each target display. Consequently, one *T* junction stimulus was shown in all four positions of the target displays, only one of which was the target. Thus, participants could not use the target’s second line’s color to efficiently guide search because searching for each of these particular colors would have misguided attention toward a distractor in two-thirds of all trials. In turn, this renders a conjunctive search for the positive target color and suppression of the color of the (negative) distractor a more efficient search strategy.

### Stimuli and procedure

**Figure 1:**
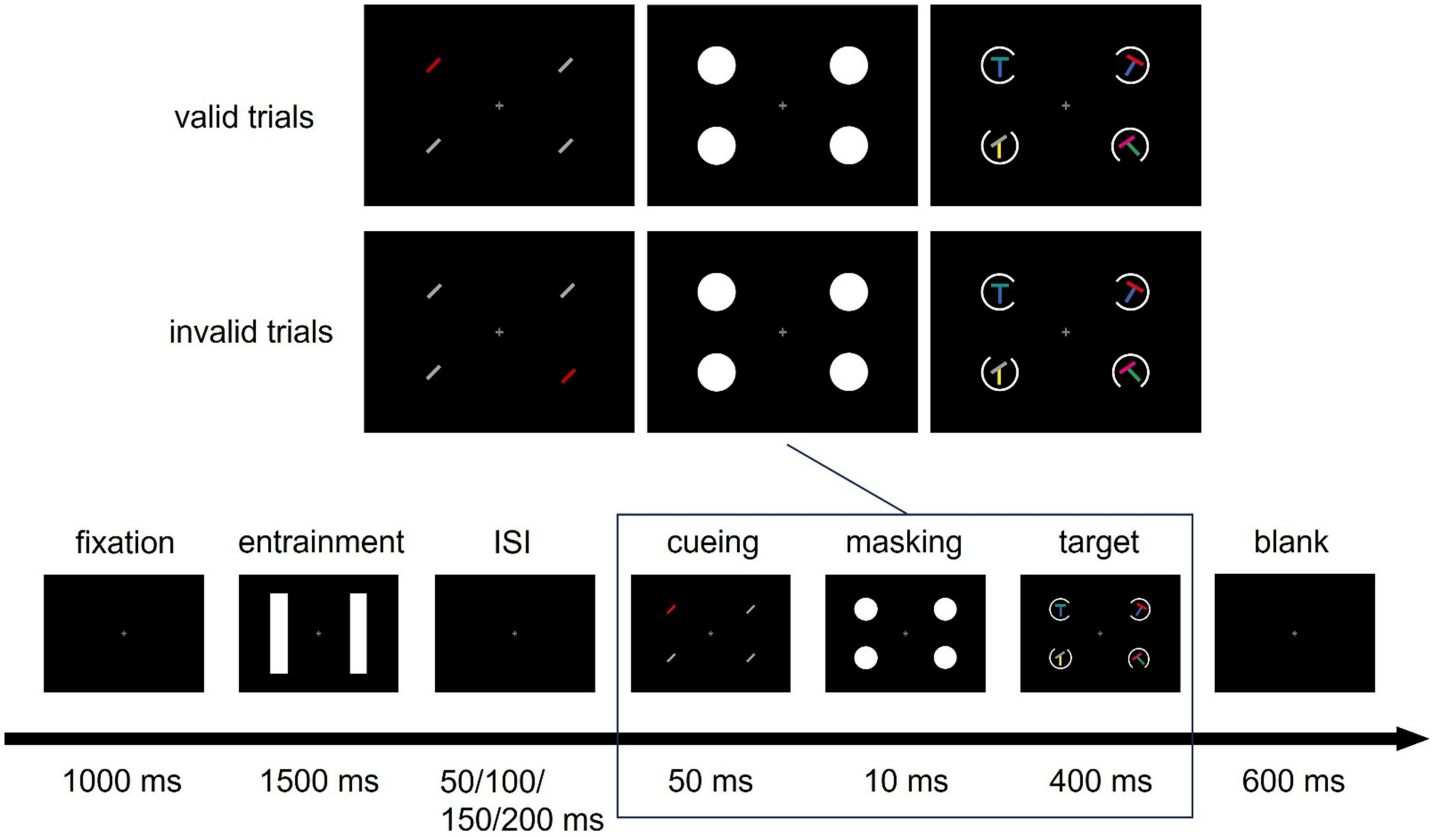
Sequence of Events in a Trial. Example of a valid and an invalid trial. In the cueing display, a negative cue with a to-be-suppressed feature (the color red) was presented. Participants had to search for a target defined by a blue base bar and a non-red top bar (in both example trials the target is at the top left) and report the orientation of a gap in a circle around the target using the arrow buttons (here, the gap is on the right). One of the bars in the entrainment display flickered rhythmically (at 10 Hz), one flickered non-rhythmically. ISI: Inter-stimulus interval.

Figure 1 shows an exemplary trial, with the relevant stimuli in the target display. Every trial started with a fixation display for 1 s, consisting of a black background (CIE L*a*b*, 7.8/22.7/−27.4) and a white (L* = 140, a* = 0, b* = 0) fixation cross with a size of 0.5 × 0.5 degrees of visual angle (°) in the middle of the screen. The fixation cross remained on the screen for the entire length of the trial, except for visual performance feedback between trials. The fixation display was followed by an entrainment stimulus, a train of visual flashes provided by white (L* = 140, a* = 0, b* = 0) vertical rectangles, with each flash lasting 10 ms. The shorter, horizontal side of each rectangle was 5° long, while the longer, vertical side measured 14°. In each trial, for 1.5 s, one of the rectangles flickered rhythmically at 10 Hz, resulting in 16 flashes and an interval of 90 ms between the respective flashes. The other rectangle also flashed 16 times for 10 ms during the same period, but its timings were generated randomly, with the exception that at least one blank frame between two consecutive flashes was present. The first and last flash of the non-rhythmic flicker bar were identical in time with the first and last flash of the rhythmically flickering bar (i.e., the entrainment stimulus) on the other side. Which side flickered rhythmically and which non-rhythmically was counterbalanced over the four blocks of the experiment. While half of the participants conducted a block with entrainment on the left (L) side in the first block of the first session and entrainment on the right (R) side in the second block of the first session, this order was reversed for the second session, resulting in two possible block sequences: L-R-R-L and R-L-L-R. Entrainment was followed by a blank display of variable duration of either 50, 100, 150, or 200 ms. This interstimulus interval (ISI) was determined randomly on each trial.

Following the ISI, a cueing display consisting of four lines (1.6° long, 0.2° wide) was shown for 50 ms. The four lines were presented equally spaced, one in each of the corners of a virtual square centered on the screen, with a distance of 5.6° between each line and the screen center. All four locations were, thus, covered by the entrainment bar or by the non-rhythmically flickering bar in the display before. Three of the lines in the cueing display were identical nonsingletons [gray (L* = 70, a* = 0, b* = 0) lines uniformly clockwise tilted by 45° in half of the trials, and counter-clockwise tilted by 45° in the other half]. One line was a red color singleton and, thus, distinct from the other nonsingleton lines only by its color, thereby serving as a cue (red; L* = 70, a* = 99, b* = 90). Across trials, the position of the singleton cue was pseudorandomized across all four positions and uncorrelated with the position of the target. The cue also did not inform about the response-relevant features.

After the cueing display, a masking display containing four white disks (3.5° diameter, L* = 140, a* = 0, b* = 0), presented at the same positions as the lines, appeared for 10 ms to prevent color fusion between cueing display and target display. Subsequently, the target display was shown for 400 ms: Participants had to search for a target consisting of an (oblique or orthogonal) *T* junction between two orientated lines. Instructions stressed both speed and accuracy.

**Figure A1:**
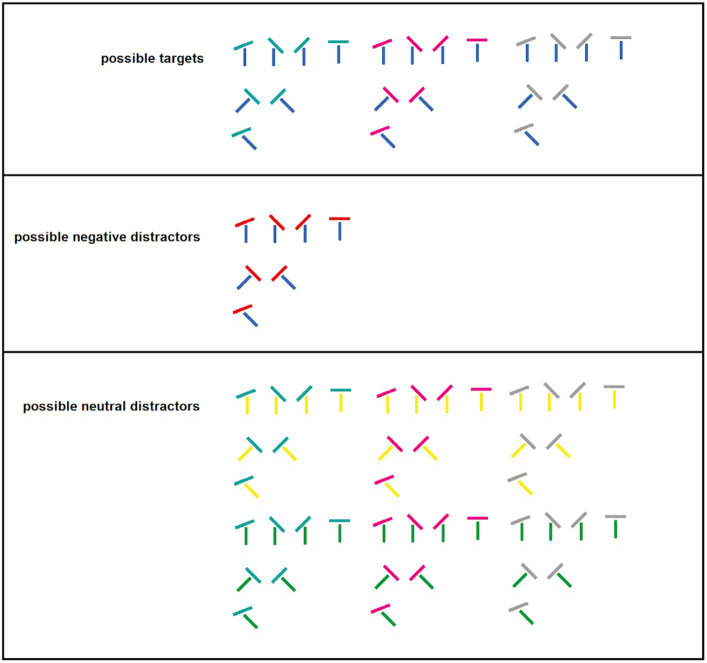
Target display stimuli. Every stimulus that could appear in the target display. The upper row shows all possible targets that could appear. The middle row lists every possible negative distractor and the bottom row all possible neutral distractor stimuli. However, only certain stimuli could be combined in target display.

In two thirds of the trials (target-present trials), one target, one negative distractor, and two further target-display distractors were presented, with one stimulus per position occupied by the lines of the preceding cueing display. Each of the four stimuli in the target display consisted of a *T* junction of two colored lines (see Figure A1, for possible combinations). The target was defined by two features: the presence of a blue (L* = 70, a* = 25, b* = −110) vertical, 45° clockwise- or counter-clockwise-tilted base line; and the absence of a red (L* = 70, a* = 99, b* = 90) top line (red distractor top lines could be horizontal or tilted by 23° or 45°). Participants were instructed to use both of these features conjunctively to search for the target. This was also the most efficient strategy for finding the target because the top line of the target was either gray, magenta (L* = 70, a* = 105, b* = −81), or cyan (L* = 70, a* = −41, b* = −20), with equal probability, and each of these three colors was present per each target display (one as a target feature, the other two in the two remaining distractors). After finding the target, participants had to report the orientation of a gap in a circle around the target by pressing the respective arrow button (for details, see below). The negative distractor shared the blue line with the target but had a red line (negative feature) that had to be ignored to find the target. Two distractors presented together with the target and the negative distractor stimulus consisted of one gray, cyan, or magenta line plus one green (L* = 70, a* = −70, b* = 67) or one yellow (L* = 70, a* = 0, b* = 73) line.

The remaining one-third of trials did not contain a target. In half of these “no-go” trials, a negative distractor was present in the following called “distractor-present condition,” while in the other half, it was not (“distractor-absent condition”). Positions becoming vacant were filled by the aforementioned distractors. These trials were included to isolate the Pd elicited by the negative feature, otherwise the ERP by the negative distractor is contaminated by a target-elicited N2pc, another event-related potential indicative for attentional capture (Eimer, 1996; Hickey et al., 2006). Furthermore, including no-go trials with or without target-similar distractor allowed us to look at cue-elicited ERPs in isolation.

In the target display, four white rings (3.5° diameter) were present: One ring in each of the four positions, surrounding the *T* junction stimuli. Each ring had a gap at one of four possible positions: at the top, at the bottom, on the right, or on the left, with each gap position occurring once per target display. Participants were required to report the gap position, which was pseudorandomized to appear equally often at each of the four locations, via the arrow buttons of a regular ‘qwertz’ keyboard. Following the target display, a fixation display was shown for up to 600 ms or until the participant pressed one of the possible answer keys. If participants responded wrongly, the fixation cross turned red for 250 ms immediately after their response, before the next trial started.

After practice trials, participants completed a total of 1,728 trials in a single session, with self-paced breaks after every 108 trials. By orthogonally combining each of the four singleton cue positions with each of the four target positions (only in target-present trials), we created valid trials, with singleton cue and target at the same position in 25% of all trials, and invalid trials, with singleton cue and target at different positions in the remaining 75% of the trials. In effect, cues were on average not predictive of the target position.

### Data recording, processing, and analysis

#### Behavior

We used RStudio (Version 1.4.1717; RStudio Team, 2021) with R (version 4.1.2; R Core Team, 2021) and the R packages broom (Version 0.7.9; Robinson et al., 2021), data.table (Version 1.14.2; Dowle and Srinivasan, 2020), dplyr (Version 1.0.7; Wickham et al., 2021), ggplot2 (Version 3.3.5; Wickham, 2016), ggrepel (Version 0.9.1; Slowikowski, 2021), rstatix (Version 0.7.0; Kassambara, 2021), and schoRsch (Pfister and Janczyk, 2016) for data analyses. Additionally, we used JASP (Version 0.16.3; JASP Team, 2022) to compute the inclusion Bayes Factor for the theoretically most important predictions, based on a Bayesian repeated-measures analysis of variance (ANOVA). The inclusion Bayes Factor is commonly interpreted as evidence in the data for including a certain predictor in the model (Hinne et al., 2020). For the ANOVAs, we used partial eta-squared ( $\eta_{p}^{2}$ ) as effect size measure (Richardson, 2011). For all further analyses, we used Cohen’s *d* as effect size standardized with the pooled within-subject *SD* and applied Hedges’ correction factor (Hedges, 1981). We used a significance level of α = 0.05 and Holm-corrected *p*-values for multiple comparisons (Holm, 1979).

We first conducted an analysis with accuracy rates (ARs) as the dependent variable. We transformed these rates using a logit transformation, which is an alternative to the arcsine transformation with greater interpretability and higher power in case of binomial data (Warton and Hui, 2011). One hundred percent accuracy rate (AR) is equivalent to 0.00 log ARs, with log ARs getting more negative with decreasing ARs. For reasons of intelligibility, we report means, *SD*s, and 95% CIs with nontransformed ARs in the results.

Additionally, we conducted analyses on participants’ reaction times (RTs). For the calculation of RTs, only correct trials (92.8% of all trials) were used. We excluded 1.02% of all responses because they were faster than 150 ms or slower than 1,000 ms. On average, we excluded 10.08% of trials (trials with a wrong answer, timeouts, and trials with too early responses) per participant (*SD* = 7.17%). For RTs, we calculated validity effects for each participant in each condition (see below for details) as invalid (cue position ≠ target position) minus valid (cue position = target position) performance, while for ARs, we did the opposite and subtracted invalid from valid performance. This ensured that better performance in valid than in invalid trials always resulted in a positive sign before the corresponding validity effect, while inverse validity effects always had a negative sign, irrespective of dependent variable.

#### Electrophysiology

The EEG signal was recorded at 512 Hz throughout the entire experiment from 132 channels using an ActiveTwo Biosemi™ electrode system (BioSemi B.V., Amsterdam, Netherlands). One hundred twenty-eight electrodes were mounted on an electrode cap according to an equiradial ABC layout, while two electrodes used as an offline reference were placed on the left and right mastoid. To monitor eye movements and blinks, the two remaining electrodes were placed 3 cm below the right eye and next to its outer canthus. EEG preprocessing was done using MATLAB (The MathWorks Inc., 2022), and the toolboxes EEGLab (version 2022.1; Delorme and Makeig, 2004) and FieldTrip (Oostenveld et al., 2011).

After resampling the data to a rate of 256 Hz and rereferencing, we applied a low-pass filter at 1 Hz and a high-pass filter at 40 Hz. We then segmented the data into 4-s epochs, from 1 s before the time-locking stimulus (the first visual stimulation burst in each trial) to 3 s after. Subsequently, we rejected faulty channels and epochs containing noise (7.18% of all epochs). Thereafter, we performed an independent component analysis (ICA; picard algorithm) to identify components related to blinks and other artifacts, such as non-ocular muscle movement, which were removed as well. Only then did we interpolate rejected channels.

We performed cluster-based permutation tests (Maris and Oostenveld, 2007) with the factors entrainment (rhythmic/arrhythmic) and condition (distractor present/distractor absent), and the amplitude of the contra- vs. ipsilateral difference wave at electrodes PO7 and PO8 (where the Pd and N2pc are known to be the most clearly visible; Sawaki and Luck, 2010) as a dependent variable in the time window between 100 and 400 ms after cue onset to see if they exhibited a Pd - a contra- versus ipsilateral positivity at posterior electrode sites that is often presumed to measure suppression (Hickey et al., 2009; but see Gaspelin et al., 2023), for a review on the Pd component including alternative accounts of its purpose), or, in case of a contra- versus ipsilateral negativity, an N2pc, indicative for attentional capture by the cue. If we found a significant time window, we followed up with nonparametric post-hoc *t* tests to isolate the effect in question. To note, applying a nonparametric approach has the advantage of detecting whether a Pd was present somewhere within this larger time window, without having gone undetected by a standard averaging approach over a predefined and narrower time window usually 115–225 ms after stimulus onset), while still adequately accounting for multiple comparisons (Sawaki et al., 2012). Trials containing a target have been omitted from the analysis, since Pd is hardly detectable when targets are present on the same side as a distractor because the Pd then overlaps with the larger ERP indicating attentional capture by the target, the N2pc. We included two types of trials without targets: (1) trials without targets, but with the negative distractor present (in the following: “distractor condition”) and (2) trials without both target and negative distractor (“cue only condition”). In both cases, we expected to find a cue-elicited Pd. We then also compared the Pd between entrainment-congruent and entrainment-incongruent sides; if the rhythmic visual stimulation boosted suppression on an electrophysiological level, we expected a greater Pd on the entrainment-congruent side.

In a second analysis, we calculated time-frequency representations for a frequency range of 1 to 25 Hz on participants spectral power (both raw and baseline-normalized; baseline window: 1 s to 0.25 s before entrainment onset) by convolving the data with a complex wavelet, constructed by multiplying the cosine and sine components at each frequency with a tapering function using a Hanning taper with a window length of 0.5 s. Additionally, we also calculated intertrial phase coherence (ITPC).

We then compared spectral power for both the time-frequency representation and the intertrial phase coherence of the rhythmic (contralateral to entrainment) versus the arrhythmic (ipsilateral to entrainment) stimulation using cluster-based permutation statistics on the respective time x frequency matrices with an alpha level of 0.05 and 10,000 permutations. For this, we selected the three channel pairs from 10 posterior electrode pairs with the highest lateralization during the entrainment period at the stimulation frequency of 10 Hz for each subject [electrodes on the left, using equiradial ABC layout notation: A8, A9, A10 (corresponding to PO7), A11, A12, A13, A14, A15 (O1), A16, A17 (approx. PO3); corresponding electrodes on the right: B5, B6, B7 (PO8), B8, B9, A26, A27, A28 (O2), A29, A30 (approx. PO4); the corresponding layout can be visited here: https://www.biosemi.com/pics/cap_128_layout_medium.jpg]. We expected a difference at the stimulation frequency or its second harmonic. In the study of visual evoked potentials, it is custom to examine responses in the harmonics because these frequencies often present clearer signals, potentially less confounded by endogenous alpha activity, particularly relevant when the stimulation frequency is around 10 Hz. We also wanted to test if these differences persisted after stimulation offset. For this, we selected a post-stimulation period between 250 ms and 500 ms after entrainment offset to (1) determine if our findings really reflected entrainment rather than an evoked brain response to flicker called the steady-state visual evoked potential (SSVEP; Norcia et al., 2015). For this, we conducted paired *t* tests between spectral power/ITPC on the arrhythmic versus rhythmic sides, correcting for three comparisons.

## Results

### Behavior

#### Accuracy rates

**Figure 2:**
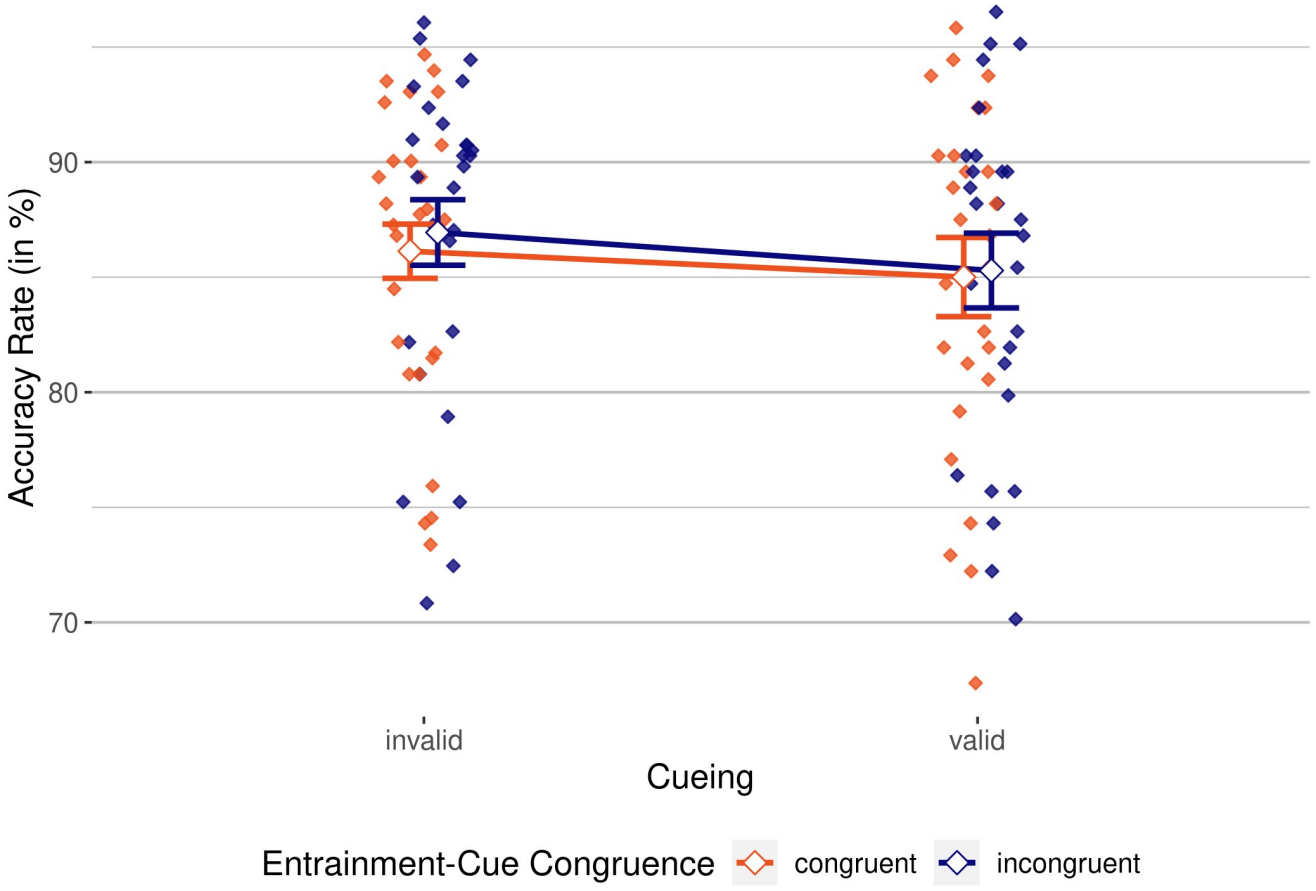
Accuracy rates by cueing and entrainment-cue congruence. Congruent in red vs. incongruent in blue. Mean Accuracy Rates (ARs), grouped by cueing condition (invalid, on the left, vs. valid, on the right) and colored by entrainment-cue congruence (congruent in red vs. incongruent in blue). Individual means are depicted as colored diamonds; averages are shown as empty diamonds. Higher scores indicate better performance. Error bars indicate 95% CIs.

For the “target present” condition, we conducted a 2 × 2 repeated-measures ANOVA, with the independent variables cueing (valid/invalid) and entrainment-cue congruence (congruent/incongruent) and ARs as dependent variable. Figure 2 shows ARs for all conditions.

We found a significant main effect of cueing, *F*(1, 26) = 8.34, *p* = 0.008, $\eta_{p}^{2}$ = 0.24, BF_incl_ = 4.17; accuracy was 1.44% worse for valid than for invalid trials, indicating an inverse cueing effect. The main effect of entrainment-cue congruence was not significant, *F*(1, 26) = 1.58, *p* = 0.220, $\eta_{p}^{2}$ = 0.06, BF_incl_ = 0.39, just like the interaction between cueing and entrainment-cue congruence, *F*(1, 26) = 0.20, *p* = 0.663, $\eta_{p}^{2}$ = 0.01, BF_incl_ = 0.31. To summarize, while ARs indicated suppression by the negative cues, the entraining stimulus did neither seem to have a modulating effect on this suppression, nor a direct (potentially lowering) effect on participants’ accuracy.

For trials without a target, we conducted a 2 × 2 repeated-measures ANOVA with entrainment-cue congruence (congruent / incongruent) and condition (distractor-present / distractor absent) on participants’ accuracy rates (note that in these trials, correct responses were equivalent to withholding a button press). The main effect of condition was significant, *F*(1, 26) = 28.08, *p* < 0.001, $\eta_{p}^{2}$ = 0.52, BF_incl_ > 100, indicating significantly better performance in distractor-absent (*M* = 99.73%, *SD* = 2.27%) than in distractor-present trials (*M* = 96.69%, *SD* = 5.08%). The main effect of entrainment-cue congruence was not significant, *F*(1, 26) = 0.23, *p* = 0.638, $\eta_{p}^{2}$ = 0.01, BF_incl_ = 0.28, just like the twofold interaction, *F*(1, 26) = 2.19, *p* = 0.151, $\eta_{p}^{2}$ = 0.08, BF_incl_ = 0.73. However, ceiling effects were clearly present, preventing further interpretation of the interaction.

#### Response times

**Figure 3:**
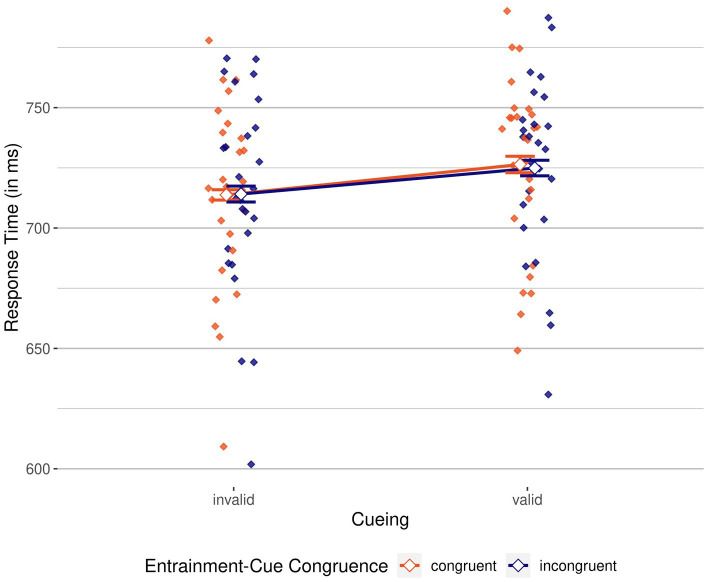
Response times by cueing and entrainment-cue congruence. Invalid, on the left, vs. valid, on the right. Mean of participants’ correct response times grouped by cueing condition (invalid, on the left, vs. valid, on the right), colored by entrainment-cue congruence (congruent, in red, vs. incongruent, in blue). Mean correct response times (RTs) are shown as unfilled diamonds, while individual mean RTs are shown as filled diamonds. Error bars indicate 95% CI.

To analyze response times, we conducted a 2 × 2 repeated measures ANOVA, with the same independent variables of cueing (valid/invalid) and entrainment-cue congruence (congruent/incongruent) on correct RTs. Figure 3 shows mean RTs for these variables.

We again found a significant main effect of cueing, *F*(1, 26) = 39.53, *p* < 0.001, $\eta_{p}^{2}$ = 0.60, BF_incl_ > 100; response times were 12 ms slower in valid compared to invalid conditions, again speaking in favor of an inverse validity effect. There was no significant main effect of entrainment-cue congruence, *F*(1, 26) = 0.14, *p* = 0.710, $\eta_{p}^{2}$ = 0.01, BF_incl_ = 0.26. The interaction was also not significant, *F*(1, 25) = 0.48, *p* = 0.496, $\eta_{p}^{2}$ = 0.02, BF_incl_ = 0.36. Evidence from RTs also corroborates the notion that while negative cues were suppressed, the entraining stimulus did not have an influence on this suppression, and also not on participants’ performance in general.

### Electrophysiology

#### Event-related potentials

**Figure 4:**
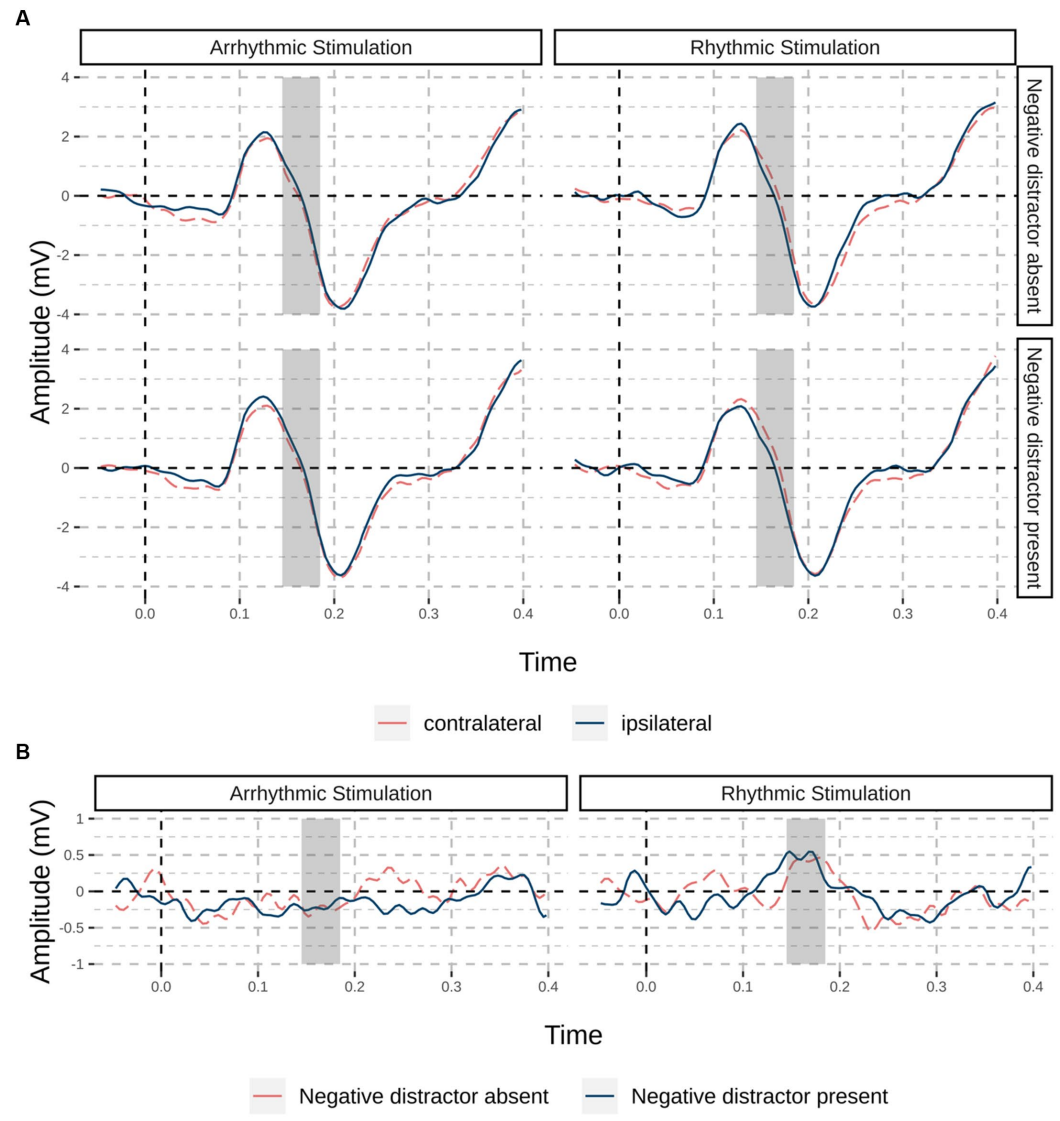
Event-related potentials for trials without a target; (orange dashed line) and ipsilateral (red solid line). **(A)** Grand average waveforms of channels PO7/PO8 split by stimulation site (rhythmic / arrhythmic) and condition, at contralateral (orange dashed line) and ipsilateral (red solid line) electrode sites; in the “negative distractor absent” condition, a cue with the to-be-suppressed negative feature, but no negative distractor was present, while in the “negative distractor present” condition, both negative cue and distractor were present. Dark shaded areas indicate the significant time window for rhythmic stimulation from cluster-based permutation testing. **(B)** Difference waves split by stimulation site and colored by condition (negative distractor absent: orange dashed line; negative distractor present: blue solid line).

For trials without a target, we were primarily interested in whether we would find a neurophysiological correlate of cue-elicited suppression (the Pd) and whether the Pd would be boosted by entrainment. Cluster-based permutation testing resulted in a significant positive cluster for the main effect of entrainment between 145 ms and 175 after cue onset, *p* = 0.033. Post-hoc tests showed that there was a significant cluster between 145 ms and 180 ms after cue onset for rhythmic, *p* = 0.023, but no significant cluster for arrhythmic entrainment. For the main effect of condition and the interaction between entrainment and condition, no significant clusters were present. Figure 4 shows the difference waves of channels contralateral and ipsilateral to the cue in the “distractor-” and “cue-only” conditions, split by whether they were caused by the arrhythmic or the rhythmic flicker.

#### Time-frequency analyses

**Figure 5:**
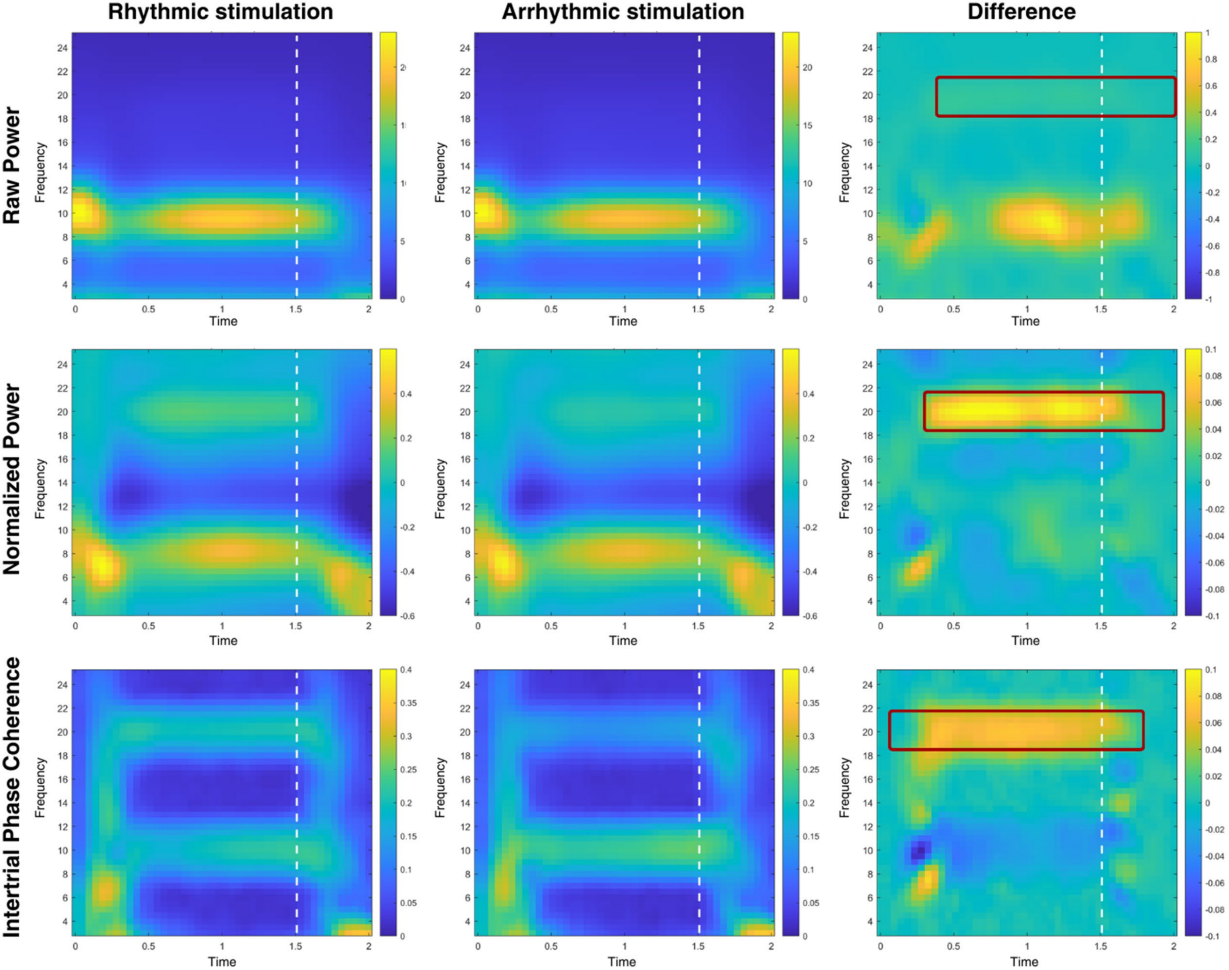
Time-frequency analysis of rhythmic and arrhythmic stimulation sites. Time-frequency plots of participants’ raw power (top row), baseline-normalized power (with the rhythmic stimulation period as baseline; middle row) and intertrial phase coherence (bottom row), shown for electrodes contralateral (labeled: rhythmic stimulation) and ipsilateral (labeled: arrhythmic stimulation) to the rhythmic stimulation. Differences in power and ITPC are shown on the right side, with a significant cluster emerging at the second harmonic of the stimulation frequency, 20 Hz (but not at 10 Hz), for all three measures. Areas marked with dark red filet rectangles represent significant clusters; visual stimulation offset is marked with white dashed lines.

Figure 5 illustrates spectral power (both with and without baseline normalization) and intertrial phase coherence (ITPC) from electrode sites referring to sites of rhythmic and arrhythmic entrainment, as well as their differences. Using cluster-based permutation statistics employing dependent-samples *t* tests, we tested for differences at 10 and 20 Hz, respectively. At 10 Hz, no significant clusters were present in spectral or ITPC contra-ipsilateral differences. At 20 Hz, we found significant positive clusters (*p* = 0.003) for non-baseline-normalized (i.e., raw power) data between 250 ms and 2,000 ms, and for baseline-normalized data between 300 ms and 1,900 ms after flicker onset indicating higher power caused by rhythmic compared to arrhythmic stimulation (*p* < 0.001). Looking at intertrial phase coherence (ITPC), a similar pattern emerged (bottom row of Figure 5): There was a positive cluster (*p* = 0.003) involving 20 Hz between 100 ms to 1,750 ms after entrainment onset, indicating higher phase coherence on the side of the rhythmic stimulation. Again, we found no significant clusters at 10 Hz.

Cluster-based permutation testing for the post-stimulation time window (250 ms to 500 ms after stimulation offset) showed significant differences for both raw power, *t*(26) = 2.78, *p* = 0.030, as well as for baseline-normalized power *t*(26) = 2.59, *p* = 0.031, but not for ITPC, *t*(26) = 0.19, *p* = 0.850.

## Discussion

In line with our expectations, we found significant inverse validity or cueing effects (i.e., better performance for invalid than for valid trials) for negative cues as an indicator of suppression. Previous research (Forstinger et al., 2022, as well as our pilot studies) has shown that these inverse cueing effects only occur with negative cues, carrying a to-be-suppressed color, whereas irrelevant cues, standing out by their task-irrelevant orientation, do not lead to any significant inverse cueing effects. In the domain of electrophysiology, evidence for suppression of the negative feature was corroborated by the presence of a cue-elicited Pd (only present on the rhythmically entrained side). This was a cue-elicited Pd because the distractor position was realized orthogonally to the cue position. Furthermore, spectral power differences between rhythmic and arrhythmic entrainment sides persisted well beyond the stimulation period suggested that endogenous neural activity was modulated through rhythmic visual stimulation, however only at the second harmonic (20 Hz) of the stimulation frequency. Rhythmic visual stimulation did not seem to impact inverse validity effects, failing to deliver behavioral evidence for a boost in suppression performance. Because inverse validity effects did not differ between entrainment-congruent and -incongruent conditions, we, believe that cue-elicited Pds were not critical for behavioral suppression. Thus, the stimulation may have facilitated the occurrence of the Pd (see Redding and Fiebelkorn, 2023), but the Pd might not be an entirely unequivocal reflection of behavioral suppression (Forschack et al., 2022; Gaspelin et al., 2023).

### Inverse cueing effects

We were able to show that performance was worse in valid than in invalid negative-cue trials and that, therefore, only targets presented at the position of a preceding negative cue were suppressed. Hence, our results support the notion of suppression of negative features (Chelazzi et al., 2019; Forstinger et al., 2022). One possible interpretation of this finding is that participants adopted an attentional control setting (ACS) to suppress the negative feature – a rejection template (Arita et al., 2012) – and applied it to the target-preceding cue if the cue happened to match the control setting (see note change Folk et al., 1992; Folk and Remington, 1998). In general, such top-down control effects have been attributed to working-memory (WM) representations, where ACSs could be maintained (e.g., Carlisle et al., 2011; Kerzel and Witzel, 2019). In line with this possibility, Forstinger and Ansorge (in press) observed that red cues elicited both inverse validity effects and regular validity effects (with advantages in valid compared to invalid conditions) depending on whether a current trial required a negative search for the absence of the color red or a positive search for the presence of the color red in the target. Of course, in cases such as ours, it is equally possible that participants hold their template in long-term memory (Carlisle et al., 2011). This is possible because the to-be-suppressed feature or the fate of this feature (i.e., it was to be suppressed or to-be-searched for) did not change across the course of the entire experiment. Many researchers believe that proactive memory-based suppression is limited to say the least (Beck and Hollingworth, 2015; Becker et al., 2015; Cunningham and Egeth, 2016). For example, informing participants on each trial about an irrelevant distractor color in advance of a search display sometimes increased rather than decreased target search times, implying that it was not possible for the participants to use WM feature templates for rejection to proactively suppress a stimulus with a feature matching such a hypothetical rejection template (Cunningham and Egeth, 2016; see also Wang and Theeuwes, 2018, for corresponding evidence regarding distractor positions).

In line with the possibility that our participants could have delegated attentional control to a long-term memory (LTM) representation (Carlisle et al., 2011), a number of studies showed that participants are able to learn the features that consistently define distractors and to suppress them more successfully as a consequence of such learning (e.g., Vatterott and Vecera, 2012; Stilwell et al., 2019). In principle, the same type of suppression based on a match between cue features and a rejection template, now, however, residing in LTM, could account for our findings. The effect could also result from a more automatic or non-strategic down-weighting of synaptic transmission in LTM neurons sensitive to the negative features alone (Cooke et al., 2015) rather than having to be based on a literal LTM “control setting” (which implies some kind of awareness and strategic control, factors that could have allowed the present stronger suppression of the negative compared to the irrelevant features).

A second possibility is that our instructions invited, if not even required, proactive suppression much more than many prior studies. In the present study, it was easier for participants to search for the target via a conjunction of the positive and negative feature, rather than to search for the target via two positive features because the second target color was not known in advance, and searching for a second positive color would have misdirected attention to a distractor in the majority of trials. In contrast, in prior studies, the usage of a negative template was typically not that beneficial (Moher and Egeth, 2012; Becker et al., 2015). Thus, the incentive to incorporate a rejection template, even in WM, might have been higher in the present than in many past studies. Related to this point, some evidence indicates that participants set-up and use proactive templates for rejection when a task is sufficiently difficult. Conci et al. (2019) found that informing about a to-be-suppressed negative distractor feature facilitated search, but only if target-distractor similarity was high and visual search, thus, difficult. Therefore, participants might trade the cost of a more demanding template for rejection against the overall benefits that could be achieved by its application. This could also explain our current findings, as search for a target defined conjunctively by the presence of one positive feature and the absence of one negative feature is relatively demanding in comparison to single-feature or singleton search tasks that are typical of prior research (e.g., Stilwell et al., 2019; van Moorselaar and Theeuwes, 2021).

One might assume that inverse validity effects for negative cues might have arisen due to rapid attentional disengagement after initial attentional capture (Moher & Egeth, 2012). We can rule out this possibility for a variety of reasons. First, our task properties, namely the usage of a consistent negative distractor feature as well as its maintenance during the experiment clearly promote proactive suppression (Braver, 2012; Noonan et al., 2016). The setup of the negative template itself was implicitly encouraged by the task because knowledge of the negative color was crucial to achieve optimal task performance. Moreover, we found electrophysiological evidence for cue-elicited suppression, in the form of a Pd, albeit only in case of rhythmic stimulation. This finding will be further discussed below in the section on “Alpha Entrainment.”

Theoretically, it is possible that capture by the cue preceded a cue-elicited Pd, as predicted by theories of reactive suppression (Moher and Egeth, 2012). Importantly, however, we did not find any N2pc evidence for the capture of attention by the cues eventually preceding the N2pc. This is in line with human neuroimaging data obtained by Reeder et al. (2017) that also speak for proactive rather than reactive suppression when negative cues are used: The authors found differences in preparatory activity for negative compared to neutral cues, with negative cues leading to lower activation in visual cortical areas than neutral and positive cues. Likewise, a behavioral study by Arita et al. (2012) found that setting up negative templates with the intention to ignore information can lead to search benefits, possibly by allowing proactive attentional suppression of visually distracting information. Again, electrophysiological evidence examining the time course of attentional deployments by Carlisle and Nitka (2019) corroborated this view, while Zhang et al. (2020), in another electrophysiological study, have demonstrated attentional guidance by negative templates toward potential targets during the early stages of visual search, contradicting key assumptions of the search-and-destroy hypothesis.

While our data strongly supports the presence of proactive suppression of the negative features, there are two potential alternative explanations of our findings. First, one may argue that the inverse validity effect might be due to the comparatively low probability that the target appeared at a cued location (25%), inviting participants to orient their attention to the remaining three locations. This would then in turn lead to a similar inverse validity effect because participants would have to disengage their attention from one of the three uncued locations to the target location when the target is shown at the cue’s position, rather than suppressing the cued position. However, this mechanism would also apply to top-down matching cues with a positive – searched-for – feature, for which, in a variety of experiments, no such effects were observed. Instead, cues with a color that matched an attentional control setting for target search either elicited a positive cueing effect (for a review, see Büsel et al., 2020) or no cueing effect at all (e.g., Forstinger et al., 2022). Attentional capture by context (here, nonsingleton) elements is also unlikely in light of other results (Forschack et al., 2022).

In general agreement with this possibility, the ability to proactively suppress distractors is subject to inter-individual differences (Mazaheri et al., 2011). Individuals with more attentional control are better at proactively avoiding distraction. Proactive suppression is in many situations the optimal strategy, but is relatively costly in terms of metabolic expenses and cognitive demand (Braver et al., 2007; Cai et al., 2011) if the information has to be maintained in working memory. Although we observed some inter-individual variance, with cueing effects for to-be-suppressed cues ranging from significant positive cueing effects (5 ms) to inverse cueing effects of −34 ms, inverse cueing effects for most participants were between −5 and − 30 ms.

### Modulation of Pd and beta, but not alpha activity by rhythmic stimulation

The findings from our electrophysiological data show that neural activity at 20 Hz (the second harmonic of the stimulation frequency) was indeed modulated, even impacting activity well after stimulation offset; however, we found no evidence for the entrainment of endogenous alpha oscillations through the 10-Hz flicker. To start with, our method (and duration) for inducing entrainment in alpha activity was similar to the one used by Spaak et al. (2014), who achieved entrainment using presentation of rhythmic visual stimuli on one side and arrhythmic stimuli on the other. Nevertheless, our results, although partially interpretable in a similar direction, were quite distinct from those obtained by Spaak et al.

To start with, significant difference clusters between rhythmic and arrhythmic stimulation were only present at 20 Hz, but not at 10 Hz. Concurrently, intertrial phase coherence differences were significant at 20 Hz, but not 10 Hz in favor of the rhythmic flicker. In case of power, these differences persisted up until 500 ms after the offset of the entraining stimulus, speaking for entrainment, in line with results from Spaak et al. (2014); however, in case of ITPC, this was not the case. The nonsignificant differences at 10 Hz (as opposed to 20 Hz) speak against the entrainment of endogenous alpha oscillations due to the rhythmic flicker. However, it is possible that endogenous alpha activity was modulated by both rhythmic and arrhythmic flicker, as ITPC at 10 and 20 Hz increased as a response to both, without significantly differing. To note, the jitter of the arrhythmic flicker was set to ±40 ms, with an equal presentation time of the first and the last flickering stimulus in each trial, and the same number of flickering stimuli for both. It also may have been the case that endogenous alpha was enhanced by flicker in a bottom-up manner, and that this was more the case for the rhythmic flicker, as its amplitude followed a regular course; however, it is also possible that participants’ upregulated endogenous alpha in a more top-down manner to suppress both regular and irregular flickers, but that the predictability of the regular flicker resulted in a more prominent upregulation. Finally, the arrhythmic pattern may have disrupted endogenous alpha (evident in its 20-Hz harmonic). The missing post-entrainment differences in intertrial phase coherence may point to one of the two latter possibilities, but our research cannot answer this question unequivocally. In our opinion, at least the notion that the differences come from a purely evoked potential like the steady-state visual evoked potentials (SSVEPs) can be ruled out because the SSVEP would not have produced prominent differences in a late post-entrainment period like in our case.

Another explanation for not finding decisive evidence for the entrainment of endogenous alpha oscillations may lie in the selection of a one-for-all frequency of 10 Hz, that may not induce alpha entrainment for all participants, and thus, not be optimal to flesh out alpha’s behavioral effects. Relating to the specificity and effectiveness of visual alpha entrainment, Gulbinaite et al. (2017) found that effective alpha frequencies varied between individuals (see also Notbohm and Herrmann, 2016). Under this perspective, one particular alpha frequency for entrainment may not fit and, thus, may not support alpha-based functionality in all our participants.

In regard to the potential boosting effect of the alpha flicker on suppression, we found that a cue-elicited Pd was present under rhythmic, but not arrhythmic stimulation conditions. The fact that the Pd was observed for cues presented at the alpha-congruent side only, speaks for a role of alpha in suppression (Kelly et al., 2006; Hickey et al., 2009). However, whether or not the cue was presented on an alpha-congruent side did not have any no significant influence on behavior, with inverse cueing effects being indifferent between these conditions. The same was true of alpha power, which was not showing significantly entrained increases beyond the duration of the stimulus itself. Whether the found activity boost in the beta range, at 20 Hz, that was found at alpha-congruent locations and that outlasted the entrainment stimulus had any bearings either as a persistent consequence of the alpha entrainment or as a supporting characteristic for the Pd has to be confirmed in future research. In the current study, this was definitely not what we expected to see in the first place.

To note, a study by de Graaf and Duecker (2021) also showed no influence of lateralized entrainment on stimulus discrimination performance. These authors presented flashes (rhythmic or non-rhythmic) only on one side in every trial. As pointed out by the authors, the flash train therefore might have acted as a spatial cue drawing attention to the cued hemisphere, leading to alpha desynchronization (opposed to alpha entrainment leading to alpha synchronization). In our case, however, this was made impossible by using bilateral flicker (rhythmic on one side, non-rhythmic on the other side). It, thus, may be helpful to adjust the flickering stimuli to the individual alpha frequency in future studies to see if this impacts behavioral indices of suppression. One additional limitation of our study is that we did not measure eye movements to control central fixation throughout the entrainment phase, ensuring a lateralized effect of the entraining stimulus. Thus, it would be interesting to study more systematically how cueing effects are modulated if alpha attracted or repelled the eyes.

### Effects of entrainment frequency and phase

In the current study, we did not differentiate between the distinct phases of entrainment. Mathewson et al. (2010), for example, found that following visual entrainment in the alpha range, the likelihood of detecting a near threshold visual stimulus is significantly increased when it appears at a time point when the next event (or one of the next events) in the preceding rhythmic sequence is (or are) expected. However, other research shows that this enhanced target detection can be delayed (or shifted) by phase, so that behavioral performance appears maximized at anti-phase (e.g., Spaak et al., 2014). Here, we used four different ISI intervals between entrainment and cue (100 / 200 ms: in phase; 50 / 150 ms: anti-phase) that varied pseudorandomly, so that both in-phase and anti-phase (10 Hz rhythm) ISIs were presented equally often; however, due to the complexity of the design, an analysis with the additional factor phase would result in significantly less trials per cell and, therefore, would likely be underpowered. It would be intriguing to see how manipulations of phase of the entraining stimulus modulate cueing effects by the different cue types. For this purpose, more fine-grained inter-stimulus intervals between entrainment stimulus and cue could be used to also cover the areas between in-phase and anti-phase, to study the phase-dependency of the rhythmic modulation of perception and suppression of visual input in even greater detail. In any case, we took an explorative glance at the data, with no visible effects of phase. Quantitatively, performance seemed to suffer at the longest (200 ms) interstimulus interval between entrainment offset and cueing display, but this was not true for the ISI of 100 ms, which was identical in phase angle also to the study of Spaak et al. (2014), who demonstrated evidence for cyclical changes in behavioral performance (but see Pomper et al., 2023, for results from rhythmic stimulation without such cyclical changes).

Another potential influence of our manipulation concerns temporal expectancy effects. Typically, temporal expectancy effects are beneficial and, thus, to the degree that temporal expectancy itself profited from a higher rhythmicity, processing of stimuli ipsi- rather than contralateral to the alpha entrainment stimulus should have been facilitated. However, this was not found. A reason might be that the time points of the first and the last of each of the single flash phases of rhythmic entrainment and non-rhythmic control stimuli were the same, mitigating a influence of different temporal expectancy effects.

This does not mean that temporal expectations were without influence (Mathewson et al., 2012). Temporal expectations were possible, and they might have been critical for our observed physiological alpha effects in a more nuanced way. One possible mechanism of how temporal expectations could have been critical for alpha effects in the EEG is that anticipation of events can facilitate the subsequent alignment of neuronal ensembles so that sensitivity is maximized for events and task-relevant objects (Nobre and van Ede, 2018). When looking at the time-frequency plots, it is apparent that such temporal expectations indeed took place in our case, as increases in alpha activity were present in the last 500 ms before rhythmic visual stimulation onset. While the outcome of such effects can be similar to that of entrainment by rhythmic stimulation, the exact mechanism is not the same (because cortical oscillations would not be entrained by an external zeitgeber; Lakatos et al., 2019). Numerous studies (e.g., Los and Heslenfeld, 2005; Rohenkohl and Nobre, 2011; Breska and Deouell, 2016) found such anticipatory effects. Although it can be hard to disentangle the effects of anticipation and entrainment, past work by Hickok et al. (2015) has sometimes demonstrated reliable effects of entrainment up to 1.4 s after stimulus offset, speaking against the notion that entrainment of neural populations is only possible during ongoing stimulation and that sometimes temporal expectancy effects are responsible for such findings (see also, e.g., Pomper et al., 2023). On a side note, the results obtained by Hickok et al. also provide relevant arguments against a possible phase reset through the cue itself (for which we did not find evidence in our phase analyses, see above). Future research is needed to investigate if temporal pre-warning effects might be supportive or even necessary preconditions for alpha(–entrainment) effects. An indicator for such anticipatory tuning might be a general decrease of alpha and beta power (Jensen and Mazaheri, 2010); while lower beta power (particularly around 14 Hz) decreased during entrainment, possibly indicating increased cognitive processing, there was a sharp increase in power in the entire alpha frequency range 300 ms before entrainment onset. We looked into this separately by using relative change in alpha power after baseline-normalizing with a prestimulus window of 1 s to 0.25 s before entrainment onset, but the results are not reported to keep the Results section focused on the major questions.

Finally, we did not use different stimulation frequencies, but were focusing on the role of alpha, including a non-rhythmic control condition. We only used alpha oscillations in the present study for its suspected prominent role in inhibition (e.g., Klimesch, 2012). However, future studies could look into differences between frequencies in regard to their effects on suppressing irrelevant visual input (e.g., theta).

## Conclusion

In this study, we examined if the rhythmic presentation of visual stimuli in the alpha range increases the effect of feature-specific suppression in a visual conjunction search task. In contrast to non-rhythmic visual flashes, rhythmic stimuli modulated neural responses at the harmonic of the stimulation frequency (20 Hz), but contrary to our expectations, did not seem to induce entrainment of alpha oscillations. Our results speak for feature-specific suppression in case of negative cues (as shown by inverse validity effects), with electrophysiological indices of this suppression modulated by rhythmic visual stimulation, as visible by the presence of a Pd under rhythmic, but not under arrhythmic conditions; however, this modulation did not result in behavioral changes in inverse validity effects, failing to deliver a result concurrent with our electrophysiological findings.

## Data availability statement

The datasets presented in this study can be found in online repositories. The names of the repository/repositories and accession number(s) can be found at: https://osf.io/yfh7u/.

## Ethics statement

We adhered to the Austrian Universities Act, 2002 (UG2002, Article 30 § 1), according to which only medical universities or studies conducting applied medical research have to obtain additional approval by an ethics committee. Thus, no additional ethical approval was required for the present study. The studies were conducted in accordance with the local legislation and institutional requirements. The participants provided their written informed consent to participate in this study.

## Author contributions

BS: Conceptualization, Data curation, Formal analysis, Investigation, Methodology, Project administration, Visualization, Writing – original draft, Writing – review & editing. MH: Data curation, Formal analysis, Project administration, Visualization, Writing – original draft, Writing – review & editing. MF: Conceptualization, Visualization, Writing – review & editing. UP: Conceptualization, Writing – review & editing. MaS: Formal analysis, Project administration, Visualization, Writing – original draft, Writing – review & editing. MoS: Conceptualization, Writing – review & editing. MG: Conceptualization, Visualization, Writing – review & editing. UA: Conceptualization, Project administration, Resources, Supervision, Writing – original draft, Writing – review & editing.
